# Water–air interface revisited by means of path-integral *ab initio* molecular dynamics†

**DOI:** 10.1039/d4cp02500h

**Published:** 2024-07-25

**Authors:** Fabrizio Creazzo, Sandra Luber

**Affiliations:** a Department of Chemistry, University of Zurich Zurich Switzerland fabrizio.creazzo@chem.uzh.ch

## Abstract

Although nuclear quantum effects (NQEs) have been considered on bulk liquid water, the impact of these latter on the air–water interface has not yet been reported. Herein, by performing and comparing *ab initio* molecular dynamics (AIMD) and path integral AIMD (PI-AIMD) simulations, we reveal the impact of NQEs on structural, dynamical and electronic properties as well as IR spectra of the air–water interface at room temperature. NQEs, being able to describe a more accurate proton delocalization in H-bonded system than AIMD, reveal a different structural arrangement and dynamical behaviour of both bulk and interfacial water molecules in comparison to AIMD results. A more de-structured and de-bound water arrangement and coordination are identified when the quantum nature of nuclei are considered for both bulk and interfacial water molecules. Structural properties, such as inter-/intra-molecular bond lengths, coordination numbers and H-bonding angles of bulk and interfacial water molecules here calculated, are affected by NQEs mitigating the overstructured description given by AIMD. Further evidences of an AIMD overstructured description of bulk water are in the computed IR spectra, where an increased absorption peak intensity and an increased strength of the hydrogen-bond network are alleviated by NQEs. In addition, NQEs show a valuable impact on the electronic structure of the air–water interface, reducing the total valence bandwidth and the electronic energy band-gap when passing from bulk to interfacial water. This work proves how NQEs significantly affect properties and features of the air–water interface, that are essential to accurately describe H-bonded interfacial systems.

## Abstract 2

Nuclear quantum effects on the air–water interface *via* path-integral *ab initio* molecular dynamics (PI-AIMD).

## Graphical abstract

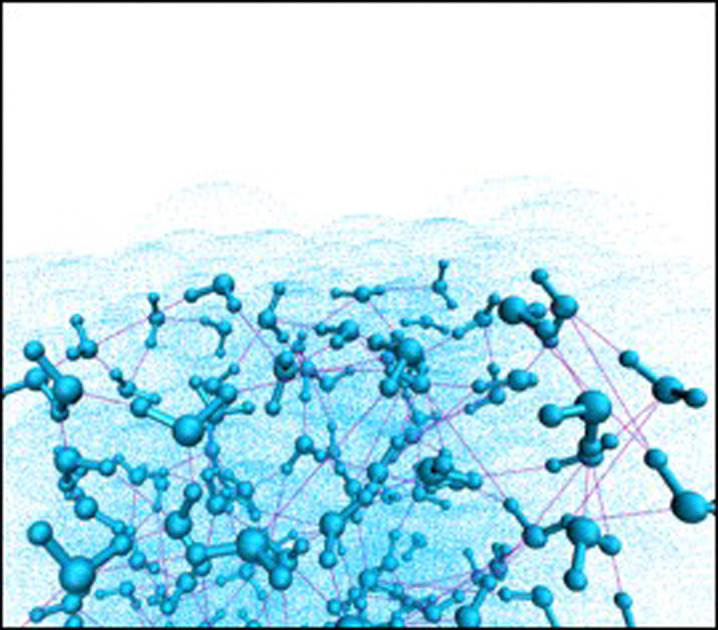

## Introduction

*Ab initio* molecular dynamics (AIMD) refers to a family of simulation methodologies able to describe the dynamical behaviour of matter within a consistent quantum mechanical framework. Density-functional-theory (DFT)-based molecular dynamics (DFT-MD) is usually also referred to as AIMD. The electronic structure is treated according to its quantum nature whereas nuclei are assumed to be propagated as classical particles, leading to a semi-classical dynamics. However, the quantum nature of nuclei significantly affects hydrogen-bonded systems and the related proton dynamics.

It has been shown that nuclear quantum effects (NQEs), such as zero-point energy (ZPE) and tunneling, can play an important role in describing waters static and dynamical properties.^1,2^ For instance, it is known that the melting point of heavy water (D_2_O) is 3.82 K higher than that of light (H_2_O) water, and this effect is more pronounced in tritiated water (T_2_O), highlighting that quantum effects destabilize the hydrogen bonded environment.^3–5^ Proton tunneling and delocalization are at the core of these isotope effects which affect not only the structural but also the thermodynamic, spectroscopic, dynamical, interfacial and solvation properties of water.^1,2,6,7^ A review and in-depth description about nuclear quantum effects on hydrogen-bonded systems can be found, *e.g.*, in ref. 1.

First attempts of molecular simulations with quantum nuclei, based on Feynman path-integral representation by employing empirical force fields, showed that quantum fluctuations soften the structure of liquid water.^8–14^ The Feynman–Hibbs theory joint with a discretized path integral description has been also used to compute the contribution of quantum effects (free energy of quantization) to the total free energy of molecular liquids (see *e.g.* ref. 15).

The first pioneering path-integral Car–Parrinello molecular dynamics (CPMD) study by Chen, Klein and Parrinello in 2003^16^ reported structural information on both H_2_O and D_2_O. The most significant finding revealed that NQEs elongate the intramolecular O–H covalent bond, shortening therefore the intermolecular O(–H)⋯O hydrogen bond distance in water compared to results based on ‘purely’ CPMD with a classical nuclear dynamics. The fully quantum-mechanical (electronic and nuclear) treatment of water enhances the water molecule dipole moment leading to a hardening (shorter intermolecular bonds in water) of the hydrogen-bonded water environment compared to results from classical nuclear dynamics. A noticeable conclusion was that the electronic and nuclear quantum treatment lead to an overstructured liquid water in comparison to CPMD simulations with classical nuclei (at the same temperature). Such a kind of simulation has already generated an overstructured liquid and then NQEs increased the discrepancy between results from simulations and experiments.

However, a later path-integral CPMD investigation by Morrone and Car in 2008^17^ on liquid water and ice provided that the inclusion of NQEs leads to a significantly less structured water environment than the corresponding one in CPMD simulation with classical nuclei. This study evidenced that NQEs significantly soften the structure of the liquid, confirming results from the above-mentioned empirical-potentials-based studies^8–14^ and a qualitative agreement with the above-mentioned experimental isotope effects. NQEs mitigate therefore the overstructuring in liquid water and thus repair (in part) the hardening of the hydrogen-bonded water environment present in CPMD simulations with classical nuclei.

A series of later computational investigations merged both conclusions leading to a concept known as ‘competing quantum effects’.^1,18–22^ NQEs on hydrogen bonded systems, such as water, can be explained by a result of two competing simultaneous phenomena: the stretching of the intramolecular O–H covalent bond (intramolecular zero point motion) and the distortion of the average water monomer geometry (intermolecular zero point energy and tunneling effects). The stretching allows the protons to be more shared (proton sharing and delocalization) between hydrogen-bonded pairs of water molecules strengthening the hydrogen bond network, overstructuring the water coordination and slowing the dynamics. The distorsion enhances the protons to spread in other directions than the axial one, leading to a distorted/bended H-bond and then a weaker hydrogen bond network, a more de-structured water and a faster dynamics of water molecules. The different contribution of these two competing effects strongly depends on the distance between oxygen atoms of the hydrogen-bonded water molecules (short hydrogen bonds = stronger bonds *vs.* long hydrogen bond = weaker bonds). This concept has been adopted during the last decade as a useful descriptor of how much impact NQEs have on the structural (and some dynamical) properties of liquid water.

From the computational point-of-view there are studies on NQEs on liquid water, however there are no investigations on other H-bonded systems such as the air–water interface. Hereinafter, with the term air–water interface we refer to the vacuum–water interface. In this study, we carry out the first path-integral *ab initio* molecular dynamics (PI-AIMD)^23–26^ study on an air–water interface, by comparing structural, dynamical and electronic properties as well as IR spectra from AIMD and PI-AIMD simulations. In this paper, AIMD and PI-AIMD have been performed for the sake of comparison having the aim of evidencing how NQEs have an impact on an interfacial H-bonded system such as the air–water interface.

Our investigations are also motivated by the reason that such kind of interfaces are complex heterogeneous systems of a vested interest for scientists in both the modeling and experimental research field, not only regarding their electronic, structural and dynamical features but also regarding their crucial role on chemical reactions that can occur at interfaces, such as *e.g.* in the photo-electrochemistry. In this context, the air–water interface and its structure are crucial, since decades, for the explanation of ‘exotic’ and still debated phenomena ranging from the (high) surface tension of water droplets,^27^ proton trapping/hopping along water wires,^28^ the atypical Pockels effect,^29^ acidity/basicity change of interfacial molecules from their values in bulk water^30,31^ to atmospheric chemical reactions at the surface of sea spray particles.^32–35^

It is worth to mention that the structure at the air–water interface is different from the bulk water structure: Hassanali *et al.*,^28^ through AIMD, evidenced water wires running parallel to the surface as the structure adopted by the first interfacial layer enhancing protons exchange between water at the interface; a series of DFT-MD investigations^36–38^ confirmed that interfacial water adopts a preferential order where the majority of interfacial water molecules (on average more than 90%) is connected by water–water H-bonds/wires oriented parallel to the instantaneous water surface forming a so-called two-dimensional (2D) H-bond network. In this context, experimental evidences based on interfacial spectroscopic techniques are in agreement with the computational findings.^39,40^ In a previous paper by one of the authors^41^ on the air–water by AIMD, it has been found that the different arrangement of interfacial water from the bulk water, *i.e.* the 2D water-wires structure at the interface, enhances proton transfers and then a protonic conductivity that is twice the one recorded in the bulk when certain values of the electric field are applied parallel to the interface.

NQEs became a mainstream feature of molecular simulations^6^ and a NQEs investigation at the air–water interface is therefore an essential key to rationalize the structural arrangement and dynamical behaviour of water at the interface with the air.

## Methods

**Fig. 1:**
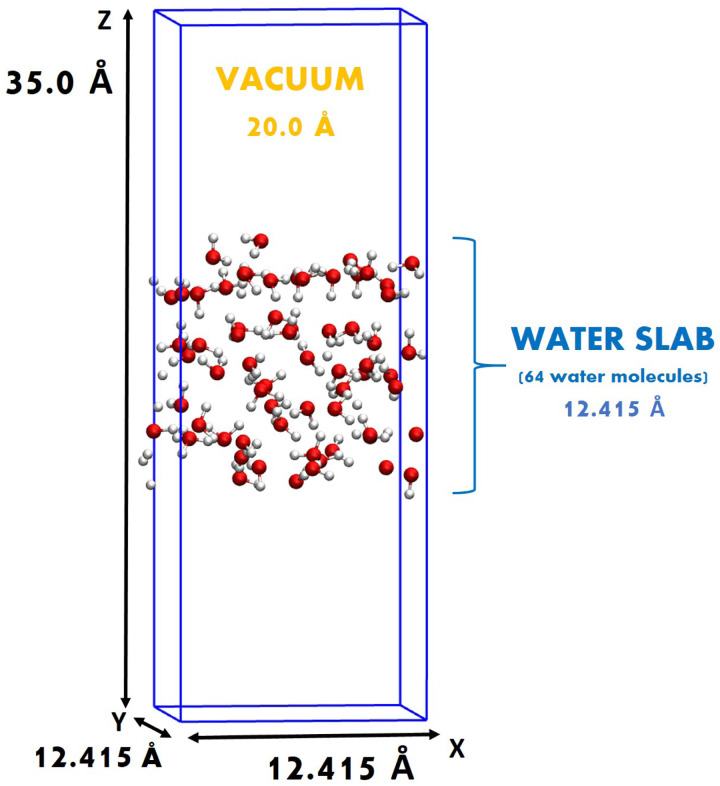
Simulation box of the air–water interface having 64 water molecules (192 atoms in total).

*Ab initio* molecular dynamics (AIMD) and path-integral AIMD (PI-AIMD)^23,24^ have been carried out at the air–water interface. By using the term AIMD we refer in this work to DFT-MD. The air–water interface has been modeled in a simulation box with side length of *x* = *y* = 12.415 Å, *z* = 35.0 Å containing 64 water molecules for a total of 196 atoms. The simulation box is periodically repeated along all three spatial directions *x*, *y*, and *z*, separated by a vacuum layer of around 20.0 Å from the replica in the vertical *z*-spatial direction. The simulation box and its dimensions are displayed in Fig. 1. See Fig. S2 in the ESI† for simulation boxes of air–water interfaces with 128 and 256 water molecules.

All simulations have been performed employing the CP2K program package.^42,43^ The AIMD with around 60 ps duration has been carried out in the Born–Oppenheimer approach by employing the Becke–Lee–Yang–Parr (BLYP) exchange–correlation functional^44,45^ supplemented with the Grimme's D3 correction^46,47^ for dispersion interactions, Goedecker–Teter–Hutter (GTH) pseudopotentials,^48^ TZV2P-MOLOPT basis set and a 400 Ry plane-wave basis set.^49^ A constant number, volume, and temperature (NVT) canonical ensemble has been adopted where the temperature has been constant at 300 K by a Nosé–Hoover chain thermostat^50^ with a time constant of 50 fs. The velocity-Verlet algorithm^51^ has been adopted with a time step of 0.5 fs. Generalized gradient approximation exchange–correlation (GGA-XC) functionals are known to underestimate structural (*e.g.* the equilibrium water density) and diffusive properties of liquid water at ambient conditions.^52,53^ The adoption of the BLYP-D3 functional is justified by extensive evidences that such a functional, when dispersion corrections are taken into account, allows one of the most reliable (among the standard GGA-XC functionals) agreement with the experimental findings.^54,55^

The PI-AIMD of around 55 ps has been carried out by means of the PIMD method^56^ as implemented in the CP2K program package. The AIMD for the electronic part has the same settings as described above. NQEs are introduced by combining the ring-polymer molecular dynamics (RPMD) approach with the generalized Langevin equation thermostat (PIGLET) method,^25^ using six beads per atom. The choice of six beads per atom relies on previous investigations^2,24,25^ where it has been shown that such a thermostating technique (PIGLET), by smoothing and accelerating the convergence, allows to reduce the number of beads needed for mapping the classical system onto the quantum one leading to an equally reliable description but a significant computational saving. An imaginary time-step of 0.5 fs has been employed for each bead of the PI-AIMD. Noise Matrices (*i.e.* matrices of the parameters for the generalized Langevin equation) used for the PIGLET colored noise thermostat here employed were taken from ref. 25 and 57. When not explicitly stated, all results refer to the centroid of the ring polymer beads. Convergence tests of both AIMD and PI-AIMD trajectories are in the ESI† in Fig. S1.

## Results and discussions

### Water density profile

The identification of a liquid–vapor interface is described in ref. 58 by Willard and Chandler in terms of an instantaneous surface fluctuating (slightly oscillating) as molecular configurations at the interface change with time. This allows to provide the water density profile with respect to the (perpendicular) distance from the instantaneous surface evaluated along the simulation time.

As previously evidenced by Willard and Chandler, the density profile shows a higher peak within the interfacial area, separated by a distinct minimum from other peaks.^58^ This peak indicates that water molecules are organized in a layered pattern near the instantaneous water–air interface. Accordingly, in previous studies,^59,60^ it has been shown that the interfacial layer is described by a higher water density in comparison to bulk water. In particular, the interfacial layer is identified by a well defined first peak at around 1.9 Å from the surface (and its minimum located at around 3.4 Å), where the average water density is around 1.5 times higher than the average water density in the bulk. Beyond the first minimum at around 3.4 Å, the water density oscillates around the bulk value of 1.0 g cm^−3^. It is therefore possible to distinguish a distinct interfacial water layer from the bulk water by the density profile but also in terms of water coordination and the average number of H-bonds for each water molecule in the interfacial layer and in bulk water.^36,59,60^

However, changes in the peak intensities of the density profile and water coordination in interfacial and bulk water have been recorded as function of the total number of the water molecules in the system and the DFT-level of theory adopted. It has been shown (at the DFT-BLYP-D2 level of theory) that at least 256 water molecules are needed for having a water density which leads to a tetrahedral-like water coordination in the bulk water (as expected) and to avoid an underestimation of the water density in the interfacial layer (expressed by an underestimation of the peak intensity in the water density profile).^36,59,60^

**Fig. 2:**
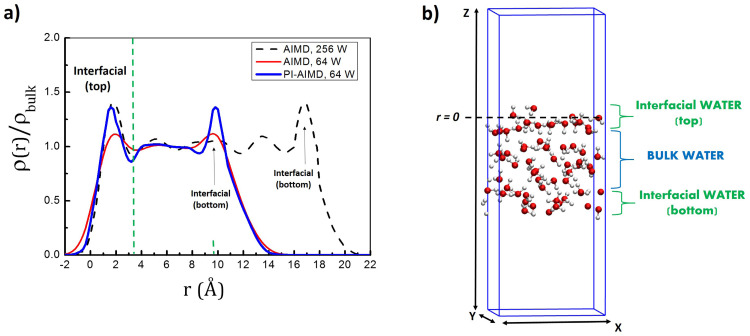
(a) Water density *ρ*(*r*) normalized with respect to the bulk water density *ρ*_bulk_. The water density is time-averaged along the simulation time and *r* = 0 identifies the position of the instantaneous surface. *r* is positive in the liquid phase and negative in the vapor phase. Few water molecules distended towards the vapor phase describe the negative water density. (b) Simulation box with 64 water molecules for the air–water interface highlighting interfacial and bulk water slab.

We report in Fig. 2 the water density *ρ* (normalized with respect to the bulk water density *ρ*_bulk_ of 1.0 g cm^−3^) as a function of the distance *r* from the surface, calculated from our AIMD and PI-AIMD trajectories with the air–water interface of 64 water molecules compared with our AIMD trajectory with the air–water interface of 256 water molecules (see Fig. S2 in the ESI† for the simulation box with 128 water molecules for the air–water interface). The DFT level of theory used in our calculations is the BLYP functional with Grimme's D3 correction.

Starting from the interface at the top (along the *z*-direction, see Fig. 2), our results show that the first peak and minima positions are close to previous findings, *i.e.* a peak at around 1.9 Å and its minimum located at around 3.4 Å from the instantaneous surface, evidencing the distinct interfacial water layer from the bulk water. The slight differences in the first peak and minima positions (within 0.1 Å) can be attributed to the statistical sampling over different simulation times (*i.e.* 60 ps for AIMD with 64 water molecules, 30 ps for AIMD with 256 water molecules and 55 ps for PI-AIMD with 64 water molecules), not affecting our conclusions.

However, the first peak intensity and the water density profile from AIMD with 64 water molecules (red line in Fig. 2) show the aforementioned underestimation in comparison to AIMD with 256 water molecules (black dotted line in Fig. 2) assumed as reference. The first peak intensity from AIMD with 64 water molecules is still larger than the one in the bulk (see the red line with an average bulk density of 1.0 g cm^−3^), evidencing still a distinct interfacial layer from the bulk water, however with an underestimated interfacial water density in comparison to AIMD with 256 water molecules. The D3 dispersion corrections adopted in our work do not really mitigate the peak intensities’ underestimation recorded also at the DFT-BLYP-D2 level in ref. 59. The same is valid for the interfacial peak attributed to the bottom interface (along the *z*-direction, see Fig. 2).

Similar conclusions are also valid for the water density profile calculated for an air–water interface of 128 water molecules. See Fig. S2 in the ESI† for the water density profile calculated for air–water interfaces with 64, 128, 256 water molecules at the AIMD-BLYP-D3 level of theory adopted in our investigations.

Noticeably, the first peak intensity and the water density profile calculated from 64 water molecules when NQEs are taken into account (blue line in Fig. 2) are quite similar to the water density profile calculated for 256 water molecules at the AIMD-BLYP-D3 level (black dashed line in Fig. 2). The interfacial water density is around 1.5 times higher than the average water density in the bulk as recorded in our AIMD-BLYP-D3 (256 water molecules) trajectory assumed as reference and in previous AIMD-BLYP-D2 (256 water molecules) trajectory.^59^ The same is valid for the interfacial peak attributed to the bottom interface. NQEs water density profile does not suffer from peaks intensity underestimations reproducing the expected water density for both interfacial and bulk water even by adopting ‘only’ 64 water molecules, instead of 256 water molecules needed for the AIMD level without NQEs.

It is worth highlighting that the aim of the paper is to compare the AIMD of the air–water interface with the one of the PI-AIMD description. Hereinafter, the comparison is done between AIMD and PI-AIMD with 64 water molecules. Besides the computational efficiency, our choice relies on the sake of consistency comparing results coming from a statistics (sampling over the simulation time) on the same number of water molecules (64). Moreover, despite the underestimation of the AIMD interfacial water density (in comparison with 256 water molecules), it is still possible to clearly distinguish interfacial water layers from the bulk water (see the red line in Fig. 2, peaks in the interfacial regions are larger and distinguished from the average bulk water density of 1.0 g cm^−3^).

The water density underestimation is one of the drawbacks of the AIMD description of the water–air interface with 64 water molecules. In the following, we highlight further differences between AIMD and PI-AIMD description of some structural and dynamical properties of the air–water interface with 64 water molecules.

### Radial distribution functions

**Fig. 3:**
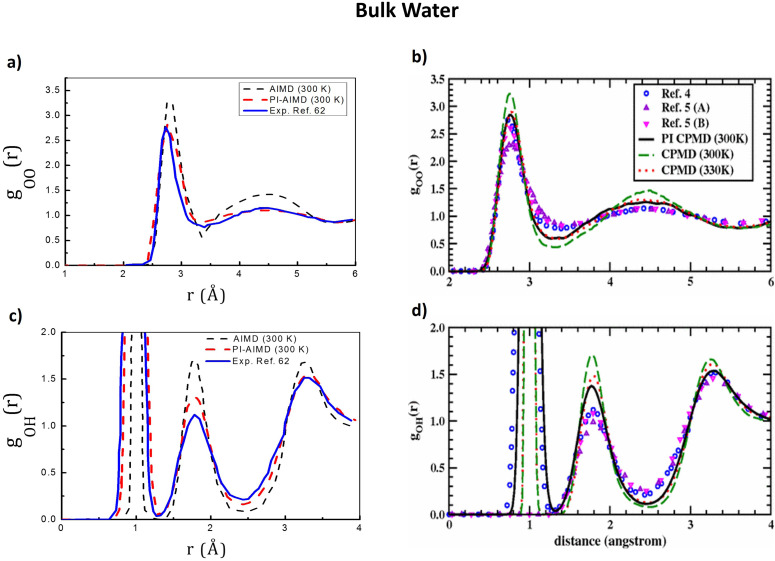
(a) O–O RDFs *g*_OO_(*r*) and (c) O–H RDFs *g*_OH_(*r*) of bulk liquid water from AIMD (at DFT-BLYP-D3 level) and PI-AIMD calculated in this work. The experimental RDFs of ref. 62 have been obtained through neutron experiments. (b) O–O radial distribution functions *g*_OO_(*r*) and (d) O–H radial distribution functions *g*_OH_(*r*) of bulk liquid water adapted from ref. 17. The legend in b is valid for d. CPMD refers to the (*ab initio*) Car–Parrinello molecular dynamics and PI CPMD refers to the path integral CPMD. Experimental RDFs of ref. 4, 5(A) and 5(B) in (b) and (d) are ref. 62, 63 and 64, respectively, in this paper.

For a better understanding of the structural arrangement of water, the radial distribution function (RDF) is a useful marker. RDFs have been evaluated for bulk water and interfacial water. As interfacial water we refer to water molecules at interfaces, *i.e.* water molecules belonging to both the upper and the bottom interfacial layers as shown in Fig. 2(b). An interfacial layer can be roughly considered as a water monolayer with a thickness of around 3.4 Å, as evidenced in the water density profile in Fig. 2(a) and in previous studies.^36–38,41,59,61^ Oxygen–oxygen (O–O) and oxygen–hydrogen (O–H) RDFs for bulk water are shown in Fig. 3(a) and (b). These latter have been calculated for the sake of comparison between AIMD and PI-AIMD (64 water molecules) in order to better understand the relative structuring of water. Our results are compared with results from ref. 17 shown in Fig. 3(b) and (d).

Looking at Fig. 3(a), the *g*_OO_(*r*) first peak position is around at 2.7–2.8 Å confirming previous known results.^55,62,65–67^ The slight difference in the first peak position of around 0.1 Å does not meaningfully alter the intermolecular (O–O) water structure and/or related properties.^2,55^ However the first peak height is also a key descriptor for the evaluation of the water structuring. The greater height of the *g*_OO_(*r*) first peak highlights that AIMD (DFT-BLYP-D3) leads to an overstructuring of the bulk liquid water, whereas NQEs are softening the bulk liquid water structure (red line in Fig. 3(a)) as also evidenced by the abovementioned studies,^8–14,17^ having a better agreement with experimental findings.^62–64,67,68^

The same is valid for the *g*_OH_(*r*) (in Fig. 3(c)) where no meaningful differences are evident for the position of peaks but their heights are affected by the NQEs’ inclusion, where the quantum delocalized character of the proton leads to a reduced discrepancy with the experimental results.^62–64,68^

Moreover, by comparing our results with results from ref. 66, we obtain a better agreement with experimental results by PI-AIMD than using AIMD (64 water molecules, cubic box of 12.445 Å side length) employing the Perdew–Burke–Ernzerhof (PBE) or strongly constrained and appropriately normed (SCAN) XC functional at 330 K (see Fig. 1 and 2 in ref. 66).

**Fig. 4:**
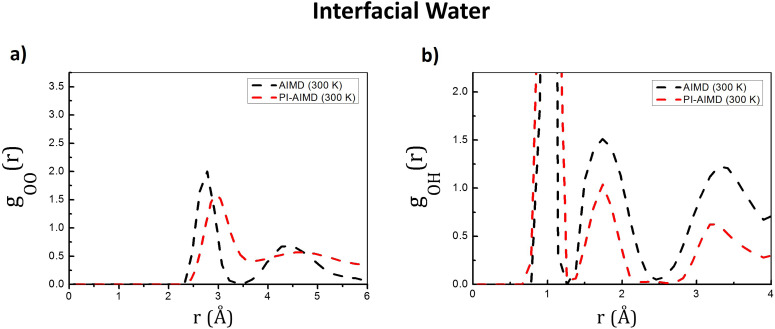
(a) O–O radial distribution functions *g*_OO_(*r*) and (b) O–H radial distribution functions *g*_OH_(*r*) for interfacial water from our AIMD (DFT-BLYP-D3) and PI-AIMD.

Our investigation has been extended to water at the interface. *g*_OO_(*r*) and *g*_OH_(*r*) have been therefore calculated for interfacial water molecules (water molecules belonging to both the upper and the bottom interfacial layers) and are shown in Fig. 4.

The first peak position of *g*_OO_(*r*) by our AIMD (DFT-BLYP-D3) and PI-AIMD is located at around 2.8 Å and 3.0 Å, respectively (see Fig. 4(a)). It is possible to observe a shift of the *g*_OO_(*r*) first peak position to a larger value when NQEs are taken into account evidencing a slightly larger distance between hydrogen-bonded pairs of water molecules. This stretching of inter-molecular interactions leads, in principle, to a slightly weaker H-bond network. This is in agreement with previous findings by PI-AIMD at bulk liquid water,^1,2,18–22^*i.e.* a more de-structured water (and accordingly a faster dynamics) is herein also evidenced for water at the interface when NQEs are considered. The height of the *g*_OO_(*r*) first peak is also affected by NQEs: the smaller height of the first peak highlights again that the NQEs inclusion mitigates the overstructured H-bond water environment (at the interface in this case) given by AIMD. The quantum delocalization of the proton when NQEs is stronger evidenced as indicated by the *g*_OH_(*r*) in Fig. 4(b).

### H-bond network

The mitigation of the overstructured water is also evidenced by the number of H-bonded water molecules. This latter is a key descriptor for the known ‘water wires’ that identify the water structure.^69^ In previous studies at the air–water interface by DFT-MD and force-field molecular dynamics (FF-MD),^37,59^ it has been evidenced that each water molecule in the bulk makes around 3.3–3.4 H-bonds in total with neighboring water molecules, where 0.8 of them are in-plane H-bonds, *i.e.* a so called intra-layer H-bond. These data are in agreement with the expected tetrahedral structure and arrangement of bulk water, and they have been estimated by defining water–water H-bonds through the criteria proposed by Galli and coworkers:^70^ O(–H)⋯O distance ≤3.2 Å and O(–H)⋯O angle in the range between 140–220°.

Conversely, at the interface, each water molecule makes around 2.9 H-bonds with neighbouring water molecules. The majority of these H-bonds, *i.e.* around 1.7–2.0 (out of 2.9), are done in plane that is interfacial water tend to maximize water–water H-bond interactions between water molecules belonging to the thin interfacial (mono)layer, leading to the so-called 2D H-bond network.^37,59^ The other one remaining H-bond is made with a water molecule ‘outside’ the thin interfacial (mono)layer, *i.e.* with the water layer below/above. One of the main results of these analyses is that water molecules at the interface make around twice the number of intra-layer H-bonds (*i.e.* in plane) than in bulk water.

Herein, by following the same H-bonds criteria^70^ we found that these values are lower for water at the interface when NQEs are taken into account. Results are compared in Table 1.

**Table 1:** Number of H-bonds per molecule averaged over the simulation time for bulk and interfacial water

Simulation	Bulk water (no. of H-bonds)		Interfacial water (no. of H-bonds)	
	Total	Intra	Total	Intra
DFT-MD-BLYP (256 W)^36^	3.3–3.4	0.8–1.0	2.9	1.7–2.0
AIMD-BLYP-D3 (64 W) this work	3.3	0.8	2.9	1.9
PI-AIMD (64 W) this work	3.3	0.8	2.2	1.4

Moreover, in order to understand how the water molecules are arranged, H-bound and oriented, we performed plane project analyses (PlProj)^71^ able to catch average in-plane structural information from our AIMD and PI-AIMD.

**Fig. 5:**
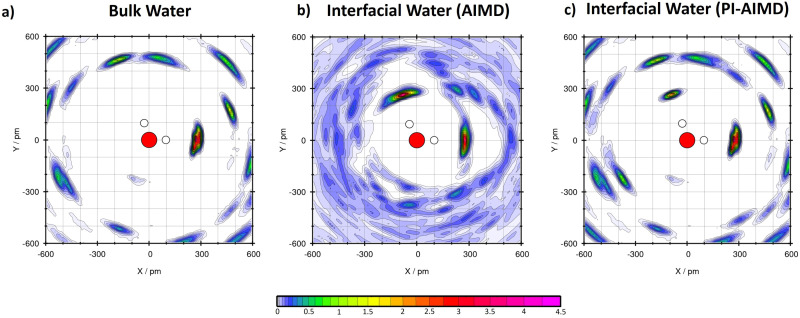
(a) Plane project analyses (PlProj) for bulk water by AIMD (BLYP-D3) and PI-AIMD in this work. The average no. of H-bonds (intra) in bulk water is around 0.8 for both AIMD and PI-AIMD (see Table 1). (b) PlProj for interfacial water by AIMD (BLYP-D3) in this work (1.9 intra H-bond on average). (c) PlProj for interfacial water by PI-AIMD in this work (1.4 intra H-bond on average). The color scale of the plot depicts the average particle density of water at the given position in the projected plane. A value larger than 1 means that water is more frequently found here than at elsewhere position, while a value below 1 indicates a depletion of water molecules at that point. *X* and *Y* indicate the in-plane spatial coordinates.

From our AIMD (BLYP-D3), we confirm previous DFT-MD results^37,59^ for bulk water and water at the interface (see Table 1). This latter in particular, having on average 2.9 H-bonds in total for each interfacial water molecule, where 1.9 H-bonds of them are done with neighboring water molecules in plane as evidenced in Fig. 5(b).

However, when NQEs are taken into account, each water molecule at the interface is able to make around 2.2 H-bonds with neighbouring water molecules (instead of 2.9 H-bonds). The majority of these H-bonds are still done in plane but having a value of around 1.4 H-bonds as shown in Fig. 5(c) (*vs.* 1.7–2 H-bonds without NQEs as shown in Fig. 5(b)). Each interfacial water molecule makes around 1.4 H-bonds with neighbouring water molecules within the thin interfacial (mono)layer, also called intra-layer H-bonds. Results are compared in Table 1.

Our results from PI-AIMD confirm the tendency of interfacial water to maximize in-plane (intra-layer) H-bonds interactions in comparison to bulk water in order to form the abovementioned 2D H-bond network^37,59^ at the interface, however with a more de-structured and de-bound water than in AIMD.

### Mean-square-displacement

**Fig. 6:**
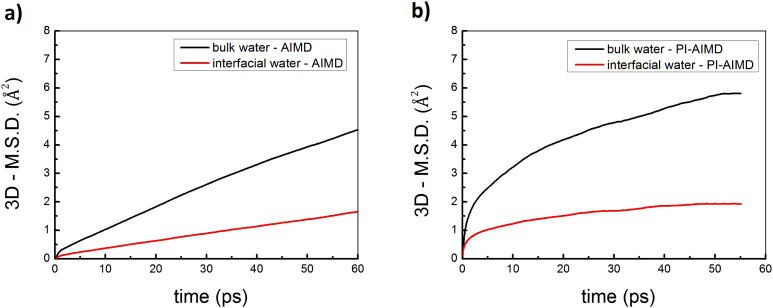
Mean-square-displacement (MSD) of oxygen O atoms along the 3 spatial dimensions (3D) by (a) AIMD in this work and (b) PI-AIMD in this work.

A more de-structured and de-bound water, as shown when NQEs are considered, involve (in principle) a faster dynamics of water molecules in the bulk as well as at the interface. The time evolution (weighted fit) of the mean-square-displacement (MSD) of oxygen O atoms along the 3 spatial dimensions (3D) is shown in Fig. 6. The MSD of the center of mass has been subtracted. The 3D-MSD have been performed for O atoms belonging to bulk and interfacial water molecules, comparing results from both the AIMD and PI-AIMD.

A word of caution is needed: well-converged diffusion coefficients could require longer time scale trajectories than the ones analyzed here, accordingly diffusion coefficients from MSD are not estimated here. However, comparing the diffusion behaviour by evaluating the MSD between bulk and interfacial water can already give us valuable insights on their respective displacement, as done in a previous paper^2^ with a simulation time lower than the ones analyzed here.

As expected, by comparing the 3D-MSD between bulk and interfacial water in both Fig. 6(a) and (b), the O atoms of interfacial water molecules have systematically a lower displacement in comparison with O atoms of bulk water molecules. This is explained by the already seen in-plane preferentially oriented H-bond network at the interface, also called H-bonded water wires or 2D-Hbond network,^36,69^ evidencing a more static/constrained dynamical behaviour for water molecules at the interface. However, by comparing the 3D-MSD given by AIMD and PI-AIMD, the displacement of O atoms is systematically larger (for both bulk and interfacial water) when NQEs are taken into account, highlighting again the more de-structured/de-bound water and accordingly a faster dynamics than in standard AIMD.

### H-bonding angles

In order to further analyze the local arrangement of water molecules, we have computed the time-average of the triplet oxygen–oxygen–oxygen angle *θ*_O–O–O_ within the first coordination shell for water molecules in the bulk and at the interface, comparing results from AIMD and PI-AIMD.

**Fig. 7:**
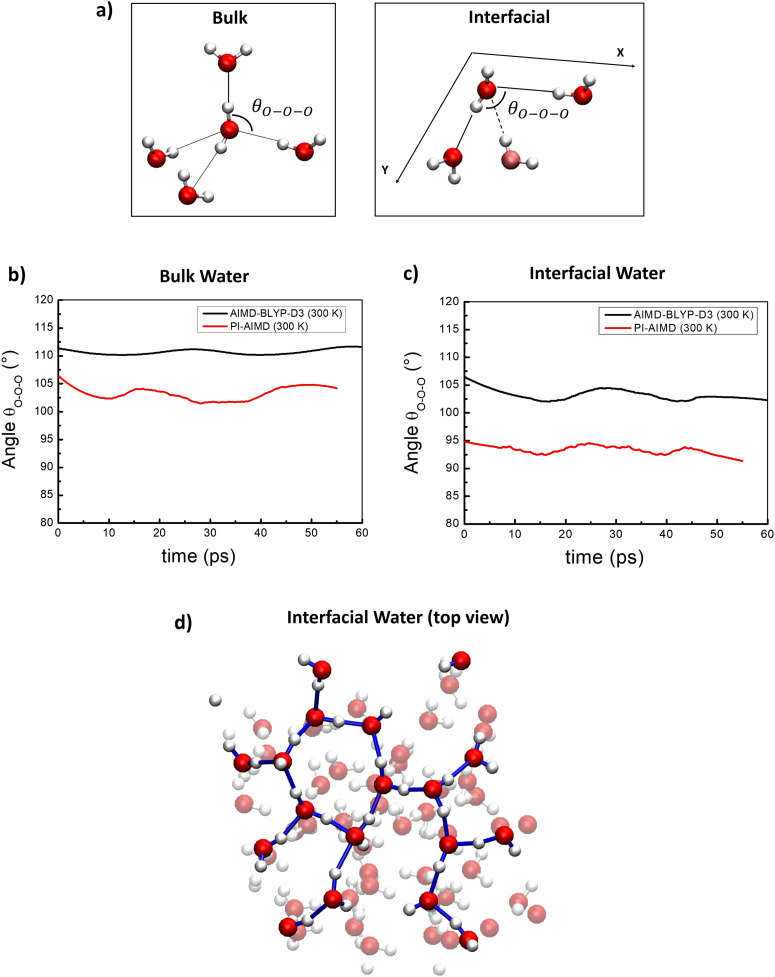
(a) Schematic view of the *θ*_O–O–O_ angle considered for bulk and interfacial water. For the interfacial *θ*_O–O–O_ angle the water molecule in faded color is not in the *X*–*Y* interfacial plane and not consider for the *θ*_O–O–O_ angle definition. (b) Time-averaged *θ*_O–O–O_ angle for bulk water. (c) Time-averaged *θ*_O–O–O_ angle for interfacial water. (d) Top view of interfacial water H-bond network from a selected snapshot highlighting the abovementioned H-bonded water wires (or 2D-Hbond network^36,69^).

To estimate the *θ*_O–O–O_ angle values, three O atoms were considered as part of a given triplet if two of the O atoms were within a cutoff distance from the third one,^66,72^ see Fig. 7(a). The cut-off distance has been chosen in order to have an ideal average O–O coordination number of 4 in the bulk as done in ref. 67, and 3 at the interface. For water at the interface, where the tetrahedral network is not anymore present, we considered the planar *θ*_O–O–O_ angle between in-plane interfacial water molecules for a better comprehension of the planar arrangement (within the interfacial water layer), see Fig. 7(a).

From Fig. 7(a), we found that at the AIMD-BLYP-D3 (300 K) level the average *θ*_O–O–O_ angle value is around 112°, close to the perfect tetrahedral angle of 109.5°, evidencing an overestimation of the local tetrahedral angle in the bulk liquid water network. The overstructuring of the H-bond network in bulk liquid water has been highlighted also by previous studies where an average *θ*_O–O–O_ angle value of around 109° has been recorded at the PBE-level at 300 K^72^ and of 104° at the PBE-level at 330 K.^66^

When the quantum nature of atoms is taken into account, the average *θ*_O–O–O_ angle value between bulk water molecules decreases to around 102.5° (see Fig. 7(b)). This latter is closer to the reference value of 100.5° recorded by joint X-ray/neutron scattering experiments,^67^ suggesting that the local bulk water arrangement is considerably more disordered/de-structured than the perfect tetrahedral coordination.

Our results further confirm that that bulk liquid water generated by AIMD using BLYP-D3 is overstructured with an overestimation of the degree of local tetrahedral order observed in experiments. However, NQEs are able to mitigate and reduce the gap between experimental results (100.5°)^67^ and computer simulations. This is also supported by the more evident oscillations around the average value of 102° (red line in Fig. 7(b)) than around the average value of 112° (black line in Fig. 7(b)), suggesting a faster dynamics related to angle distorsion when NQEs are considered, and evidencing again the overstructured estimation of the tetrahedral order given by AIMD in comparison with PI-AIMD.

Similar conclusions are valid for the interfacial water in Fig. 7(c). We found an average *θ*_O–O–O_ angle value (in plane) of 104° and 94° from our AIMD and PI-AIMD, respectively. The angle distorsion allows the protons to be delocalized and to spread towards other spatial directions than the axial one, leading to a more de-structured water.^1^ The structural H-bond network of water at the interface is shown in Fig. 7(d) as H-bonded water wires (or 2D-Hbond network) also found in previous studies.^36,69^

### Electronic properties

Further details about the water local structural coordination and the strength of its H-bond network can be obtained also by investigating electronic properties. We computed the electronic density of states (DOS) of bulk liquid water from our AIMD and PI-AIMD comparing our results with previous DOS from ref. 66 obtained by AIMD simulation with the SCAN XC functional. It has been provided in ref. 66 that the SCAN meta-GGA functional systematically improves the agreement of electronic properties with experimental results when compared to PBE and PBE+van der Waals (Tkatchenko–Scheffler^73^ dispersion correction) functionals. Essentially, SCAN is able to predict improved geometries and energies of condensed matter materials, such as bulk liquid water, by capturing the intermediate-range vdW interactions. The meta-GGA SCAN is therefore considered as one of the best in reproducing experimental results, close to results obtained through hybrid functionals.^74,75^

**Fig. 8:**
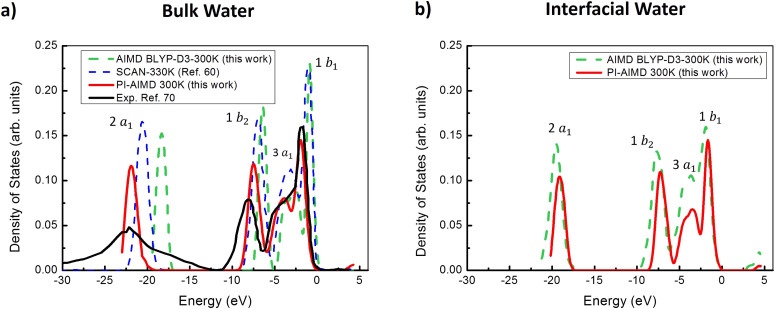
(a) Electronic DOS of bulk water from our AIMD (green dashed line) and PI-AIMD (red line) compared with DOS by SCAN functional from ref. 66 and experimental reference from ref. 76. (b) Electronic DOS of interfacial water from our AIMD and PI-AIMD. 2a_1_, 1b_2_, 3a_1_, and 1b_1_ peaks correspond to valence molecular orbitals based on the spatial symmetries of a water molecule, where 2a_1_ and 1b_1_ orbitals are related to the lone electron pairs and 1b_2_ and 3a_1_ orbitals to the bonding electron pairs. The top of the valence band is set to 0 eV.

Regarding bulk water, our DOS calculations and comparison in Fig. 8(a) highlight that the NQEs further improve the computed DOS results, mitigating more the gap with experimental DOS. The comparison of the computed electronic DOS has been done with experimental DOS from (full-valence-band) photoemission spectra.^76^

From Fig. 8(a), bulk DOS obtained using the SCAN functional is improved in comparison to our AIMD (BLYP-D3-300 K) results (especially for the 2a_1_ peak position), confirming the previous better result given by SCAN in comparison to GGA results in ref. 66.

However, when the quantum nature of nuclei is taken into account, the 2a_1_ peak position shifts 2 eV to a smaller energy compared to SCAN (red line *vs.* blue dashed line in Fig. 8(a)), in a better agreement with experimental findings^76^ in bulk water. A similar energy shift is valid for the 1b_2_, 3a_1_, and 1b_1_ peak positions leading to a further agreement with experimental DOS.

Accordingly, the valence-band energy difference Δ*E* between the 2a_1_ and 1b_1_ is around 20 eV by PI-AIMD (red line) in Fig. 8(a), 17.5 eV by AIMD (BLYP-D3-300 K), 18.98 eV with SCAN (ref. 66), 17.63 with PBE+vdW (ref. 66), confirming again the better agreement of PI-AIMD with the Δ*E* experimental value of 19.74 eV.^76^ This highlights an improved energy difference between the two electronic states and a better description of lone pair electrons. A better agreement between DOS by PI-AIMD and experiments is recorded even for 1b_2_, 3a_1_ electronic states related to bonding electron pairs.

Regarding the (electronic) band-gap energy, a value of around 4.6 eV has been obtained by PI-AIMD, that is comparable with values of around 4.51 eV using PBE,^66^ 4.32 eV using PBE+vdW,^66^ and 4.90 eV using SCAN,^66^ but still quite lower than the experimental value of 8.7 eV in ref. 77 by photoelectron emission spectroscopy (PES).

In addition, analyses of DOS have been also done for water at the interface and are shown in Fig. 8(b).

(i) By comparing AIMD (BLYP-D3-300 K) DOS results for bulk and interfacial water molecules (green dashed lines in Fig. 8(a) and (b)), it is possible to observe a slightly blue shift (to lower values) of around 0.8 eV for all peak positions given by interfacial water molecules;

(ii) By comparing PI-AIMD DOS results for bulk and interfacial water molecules (red lines in Fig. 8(a) and (b)), it is possible to observe a red shift (to higher value) of around 2.8 eV for the 2a_1_ peak position (lone electron pairs) given by interfacial water molecules. Accordingly, a reduction of the (total) valence bandwidth has been recorded (at the PI-AIMD level, see red lines in Fig. 8) when passing from bulk water to interfacial water, that is 23.5 eV *vs.* 20.7 eV width. This latter is in agreement with results of a previous study that highlighted a red shifted energy of valence lone electron pairs toward higher energies when passing from bulk to surface water;^78^

(iii) regarding the (electronic) band-gap energy, a reduction of around 1.5 eV is recorded for the interfacial water (both at the AIMD and PI-AIMD) compared to the band-gap energy in bulk water, that is more than the value of 0.5 eV band-gap reduction calculated in a previous study^78^ for the same. The decrease of the band-gap energy from bulk to interfacial water possibly enhances electron transfers from interfacial water to a possible solvent/solute placed in contact with, a crucial phenomena for reactions at aqueous interfaces.^79–84^

It is worth to mention that the electronic part in our PI-AIMD has been treated with the same computational setup used for our AIMD (BLYP-D3-300 K). However, we have shown that the PI-AIMD gives an improved and a different structural (and dynamical) arrangement of the bulk and interfacial water environment (more de-structured, longer O–O distance-see previous RDFs, angle distorsions-see previous *θ*_O–O–O_ calculations, faster dynamics-see previous MSD estimations). The different structural arrangement and dynamical behaviour given by PI-AIMD in comparison to AIMD affects even the electron distribution around atoms (that are re-arranged in agreement with the PI-AIMD structural findings and its related H-bond network, that are different from AIMD), leading to different results for the electronic DOS.

### IR spectra

The basic arrangement of water can be also investigated by spectroscopic signatures. Infra-red (IR) spectroscopy is one of the most sensitive and powerful method to detect hydrogen bonds and identify their strengths. We computed IR absorption spectra for bulk water^85^ comparing results from our AIMD and PI-AIMD.

**Fig. 9:**
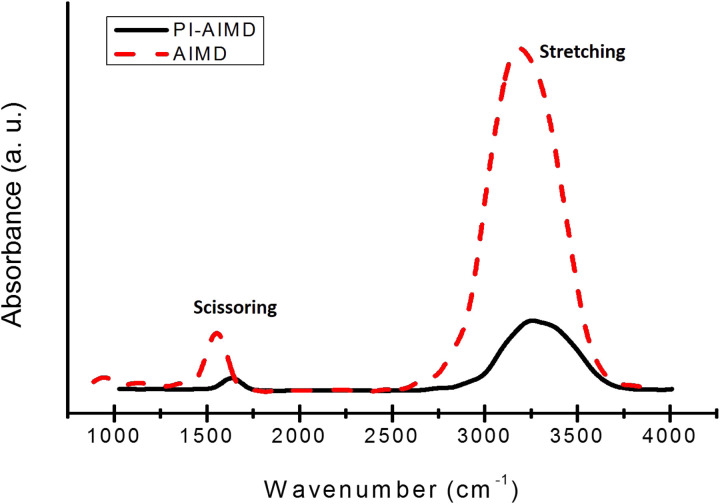
IR absorption spectra calculated for bulk water.

From Fig. 9, the peaks positions are in agreement with the known stretch and scissor mode regions in experimental studies^86,87^ for bulk water. In particular, we have a broad peak position at around 3260 cm^−1^ for the O–H stretching and at 1650 cm^−1^ for the scissoring vibration using PI-AIMD.

However, we have a red-shift to lower frequency of peaks positions by AIMD compared to PI-AIMD, having a peak position at around 3150 cm^−1^ for the O–H stretching and at 1550 cm^−1^ for the scissoring. This is in agreement with the fact that an increased strength of the hydrogen-bond network typically shifts the stretch vibration to lower frequencies (red-shift) with a significant increased peak intensity.

The intensity of an absorption band depends on the polarity of the bond and also on the number of bonds (responsible for the absorption): the absorption band with a higher polarity and more bonds has a higher intensity.^86–89^

The increased intensity and the red-shift of absorption bands estimated by AIMD highlight again the overstructured arrangement of water in comparison with PI-AIMD.

## Conclusions

We have performed AIMD and PI-AIMD simulations on the air–water interface at ambient conditions with the aim of comparing results in order to better understand how NQEs affects the air water interface. The inclusion of NQEs in hydrogen bonded systems is crucial in treating the quantum nature of nuclei and proton tunneling, which involves bond breaking and forming events that are not easily recorded by the classical treatment of nuclei. Structural, dynamical, electronic and spectroscopic properties of the air–water interface have been investigated for bulk and interfacial water. We found a systematic better agreement between PI-AIMD results and experiments, alleviating the overstructured description of bulk and interfacial water arrangement given by AIMD.

Herein, we provide evidences of the overestimation given by AIMD in describing the structural motif of bulk and interfacial water such as in the RDF, coordination number and water tetrahedrality. Not only structural properties but also dynamical behaviour of bulk and liquid water have been compared finding a more de-structured water when NQEs are considered and a faster dynamics which involves a faster bond breaking and forming. The larger intermolecular distance between H-bonded water, the H-bond angle distorsion and the ligancy motif of water molecules can support an enhanced proton delocalization in agreement with a more de-bound water arrangement when NQEs are taken into account in both bulk and interfacial water, extended the previous concept known as ‘competing quantum effect’^1,18–22^ to water molecules at the interface. All of these can give a different perspective on interfacial properties such as the surface tension and related equilibrium tension models.^90–92^

We proved that NQEs affect also the electronic structure calculated by DOS analyses in this work, finding a reduced gap between PI-AIMD results and experiments, highlighting, among others, valence-bandwidth and electronic band-gap reductions for water at the interface in comparison with bulk water DOS.

Additional evidences of the overstructured description of bulk water given by AIMD in comparison with PI-AIMD are given by computed IR spectra where the higher intensities of the absorption bands from AIMD, and the associated stronger hydrogen bond network, are mitigated by the inclusion of NQEs.

In conclusion, by using state-of-the-art PI-AIMD, we have shown that NQEs can have a significant impact on accurately describing hydrogen bonded systems such as the ones at air–vapor interfaces. Not only, this study provides new physical insight on the promising reliability of PI-AIMD for future work on modeling aqueous solutions and reaction at interfaces.

## Data availability

The data supporting this article have been included as part of the ESI.†

## Conflicts of interest

There are no conflicts of interest to declare.

## Supplementary Material

CP-026-D4CP02500H-s001

CP-026-D4CP02500H-s002
