# Supplementary material for: Water–air interface revisited by means of path-integral ab initio molecular dynamics
Source: Phys Chem Chem Phys. 2024 Jul 25;26(31):21290–302. doi: 10.1039/d4cp02500h (PMC11305098; doi:10.1039/d4cp02500h)
Supplement: CP-026-D4CP02500H-s002 [file CP-026-D4CP02500H-s002.pdf]

This journal is © the Owner Societies 2024

SUPPLEMENTARY INFORMATION

Water-Air Interface Revisited by means of Path-Integral *Ab-Initio* Molecular Dynamics

Fabrizio Creazzo[a*] and Sandra Luber[a]

[a]*Department of Chemistry, University of Zurich, Zurich, Switzerland*

* Electronic mail: fabrizio.creazzo@chem.uzh.ch

1. CONVERGENCE TEST

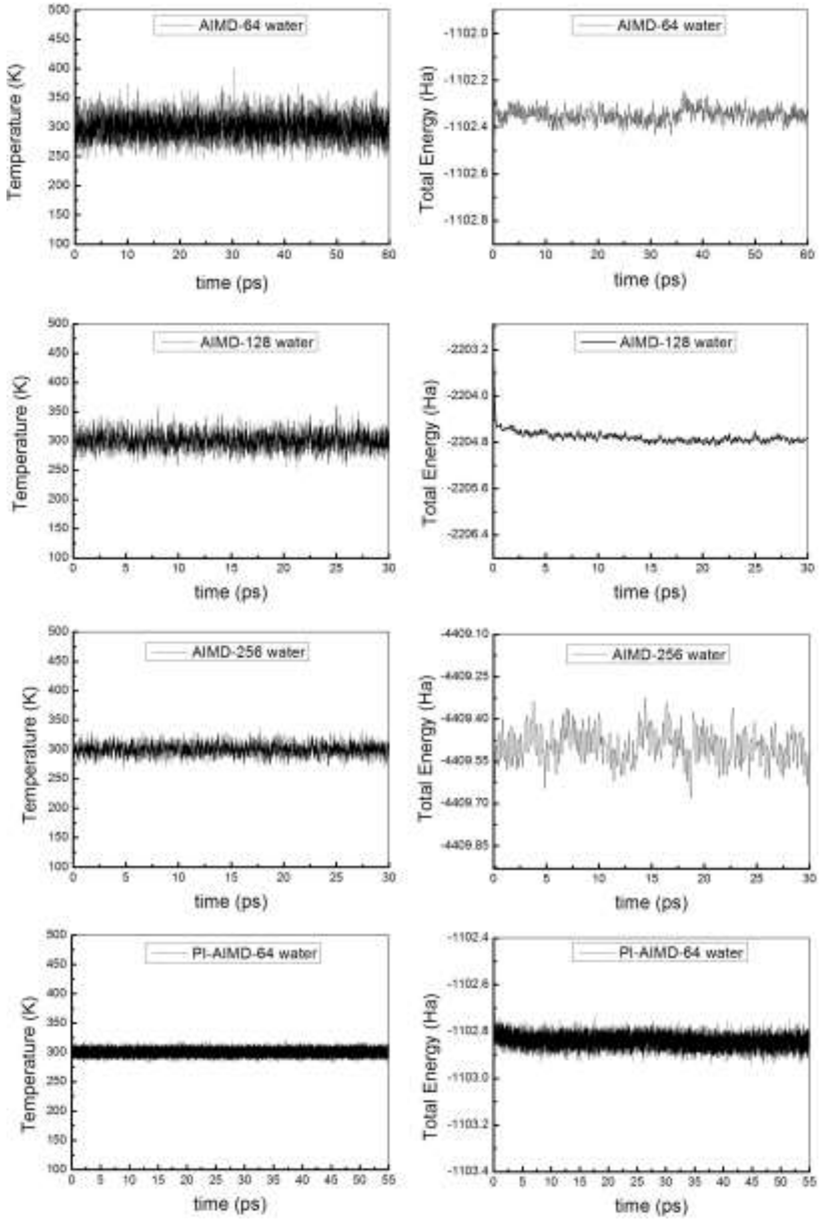

FIG. 1. *Temperature (K) and Total Energy (Ha) as a function of the simulation time for AIMD (with 64, 128, 256 water molecules) and PI-AIMD (with 64 water molecules). The NVT canonical ensemble has been adopted where the temperature has been constant at 300 K by a Nosé-Hoover chain thermostat with a time constant of 50 fs. The Velocity-Verlet algorithm has been adopted with a time step of 0.5 fs.*

2. WATER DENSITY PROFILE

Water density profile in terms of an instantaneous surface (see Ref. 58) calculated for air-water interfaces with 64, 128, 256 water molecules at the AIMD-BLYP-D3 level of theory adopted in our investigations.

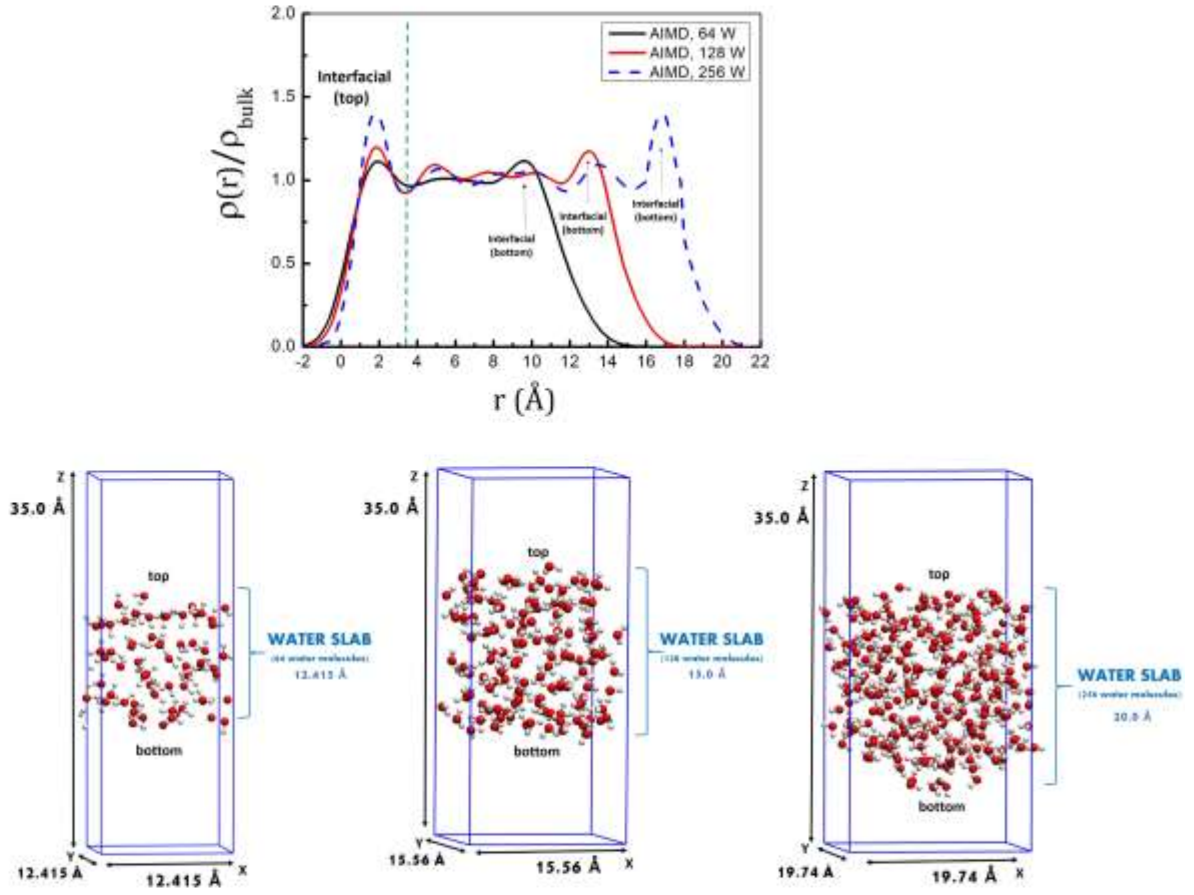

FIG. 2. *a) Water density $\rho(r)$ normalized with respect to the bulk water density ρ_{bulk}. The water density is time-averaged along the simulation time and r=0 identifies the position of the instanta- neous surface. r is positive in the liquid phase and negative in the vapor phase. Few water molecules distended towards the vapor phase describe the negative water density. b) Simulation boxes of air water interfaces with 64, 128, 256 water molecules.*
